# Anastomotic perfusion assessment with indocyanine green in robot-assisted low-anterior resection, a multicenter study of interobserver variation

**DOI:** 10.1007/s00464-022-09819-1

**Published:** 2023-01-09

**Authors:** Pernille O. Larsen, Nikolaj Nerup, Jesper Andersen, Niclas Dohrn, Mads Falk Klein, Steffen Brisling, Soren Salomon, Per V. Andersen, Soren Möller, Morten B. S. Svendsen, Hans B. Rahr, Lene H. Iversen, Ismail Gögenur, Niels Qvist, Mark B. Ellebaek

**Affiliations:** 1 Research Unit for Surgery, Odense University Hospital, University of Southern Denmark, Odense, Denmark; 2 Department of Surgery and Transplantation, Copenhagen University Hospital, Rigshospitalet, Copenhagen, Denmark; 3 Surgical Department, Colorectal Cancer Center South, Vejle Hospital, University Hospital of Southern Denmark, Beridderbakken 4, 7100 Vejle, Denmark; 4 Department of Surgery, Aarhus University Hospital, Aarhus, Denmark; 5 Center for Surgical Science, Zealand University Hospital, Roskilde, Denmark; 6 Department of Surgery, Copenhagen University Hospital, Herlev and Gentofte, Copenhagen, Denmark; 7 Open Patient Data Explorative Network, OPEN, Odense University Hospital and Department of Clinical Research, University of Southern Denmark, Odense, Denmark; 8 CAMES Engineering, Copenhagen Academy of Medical Education and Simulation, Capital Region of Denmark, Copenhagen, Denmark

**Keywords:** Anastomotic leakage, Fluorescence, Inter-observer variation, Colorectal surgery, q-ICG

## Abstract

**Background:**

Securing sufficient blood perfusion to the anastomotic area after low-anterior resection is a crucial factor in preventing anastomotic leakage (AL). Intra-operative indocyanine green fluorescent imaging (ICG-FI) has been suggested as a tool to assess perfusion. However, knowledge of inter-observer variation among surgeons in the interpretation of ICG-FI is sparse. Our primary objective was to evaluate inter-observer variation among surgeons in the interpretation of bowel blood-perfusion assessed visually by ICG-FI. Our secondary objective was to compare the results both from the visual assessment of ICG and from computer-based quantitative analyses of ICG-FI between patients with and without the development of AL.

**Method:**

A multicenter study, including patients undergoing robot-assisted low anterior resection with stapled anastomosis. ICG-FI was evaluated visually by the surgeon intra-operatively. Postoperatively, recorded videos were anonymized and exchanged between centers for inter-observer evaluation. Time to visibility (TTV), time to maximum visibility (TMV), and time to wash-out (TWO) were visually assessed. In addition, the ICG-FI video-recordings were analyzed using validated pixel analysis software to quantify blood perfusion.

**Results:**

Fifty-five patients were included, and five developed clinical AL. Bland–Altman plots (BA plots) demonstrated wide inter-observer variation for visually assessed fluorescence on all parameters (TTV, TMV, and TWO). Comparing leak-group with no-leak group, we found no significant differences for TTV: Hazard Ratio; HR = 0.82 (CI 0.32; 2.08), TMV: HR = 0.62 (CI 0.24; 1.59), or TWO: HR = 1.11 (CI 0.40; 3.11). In the quantitative pixel analysis, a lower slope of the fluorescence time-curve was found in patients with a subsequent leak: median 0.08 (0.07;0.10) compared with non-leak patients: median 0.13 (0.10;0.17) (*p* = 0.04).

**Conclusion:**

The surgeon’s visual assessment of the ICG-FI demonstrated wide inter-observer variation, there were no differences between patients with and without AL. However, quantitative pixel analysis showed a significant difference between groups.

**Trial Registration:**

ClinicalTrials.gov Identifier: NCT04766060.

Anastomotic leakage (AL) is a serious complication after low anterior resection for rectal cancer and is associated with an increased risk of postoperative mortality and morbidity, cancer recurrence, and impaired functional outcome [1–3]. In the last 3 years, the rate of clinical ALs registered in the nationwide Danish Colorectal Cancer Group’s database has been around 9% [4].

Among several factors, sufficient blood supply and oxygen delivery to the anastomotic area is crucial to ensure optimal conditions for anastomotic healing [5]. Traditionally the evaluation of blood supply has been based on the surgeon’s subjective surrogate measures such as tissue colour, mesenteric pulsation, and marginal arterial bleeding. Clinical studies have demonstrated that the surgeon**’**s intraoperative judgment in predicting AL, based on these parameters, has a low sensitivity and specificity [6]. Several other different techniques have been evaluated for a more objective evaluation of the blood supply including laser Doppler flowmetry [7, 8], near-infrared spectroscopy [9, 10], intra-mucosal pH measurements [11], and tissue oxygen tension [12] as the most common, but none of these methods has become routine in clinical use.

Clinical studies in robot-assisted and laparoscopic colorectal surgery confirm the feasibility of using indocyanine green fluorescent imaging (ICG-FI) intra-operatively [13–19], and cohort studies have shown that when taking into account whether to re-do the anastomosis or not it may reduce the AL rate by 54–67% [14, 16, 20, 21]. Two randomized clinical trials have been published [22, 23]. One of the studies showed a signifcant difference in grade A anastomotic leakage in those who had undergone a perioperative ICG evaluation compared to a control group and no difference in the other study. Software-based pixel analyses to quantify the fluorescence signal have been developed [24–26], but no cut-off values for anastomotic re-do have been provided. Animal experimental studies have shown that quantifying indocyanine green fluorescent imaging (q-ICG-FI) can be used as a surrogate measure of local bowel blood perfusion. In addition, a correlation between relative flow in the anastomotic area and anastomotic strength as assessed by stretch-tension and histological healing has been demonstrated [27–29].

Different doses of ICG have been used [30, 31]. However, there is no consensus on the optimal dose or whether this has any importance for the visual interpretation of the ICG-FI.

The primary aim of the present study was to investigate inter-observer variation among surgeons regarding their visual assessment of ICG-FI and based on this assessment, whether they would recommend a re-do of the anastomosis to prevent AL. Our secondary aim was to compare the results from both the visual and the quantitative assessment of ICG-FI in relation to the development of AL, and to investigate whether the dose of indocyanine green (ICG) had an influence on the assessment.

## Material and methods

This was a prospective study including patients undergoing robot-assisted low anterior resection with stapled anastomosis. We included patients from five different colorectal centers in Denmark from April 2017 to November 2018. Each center included between six and 22 patients.P

**Fig. 1:**
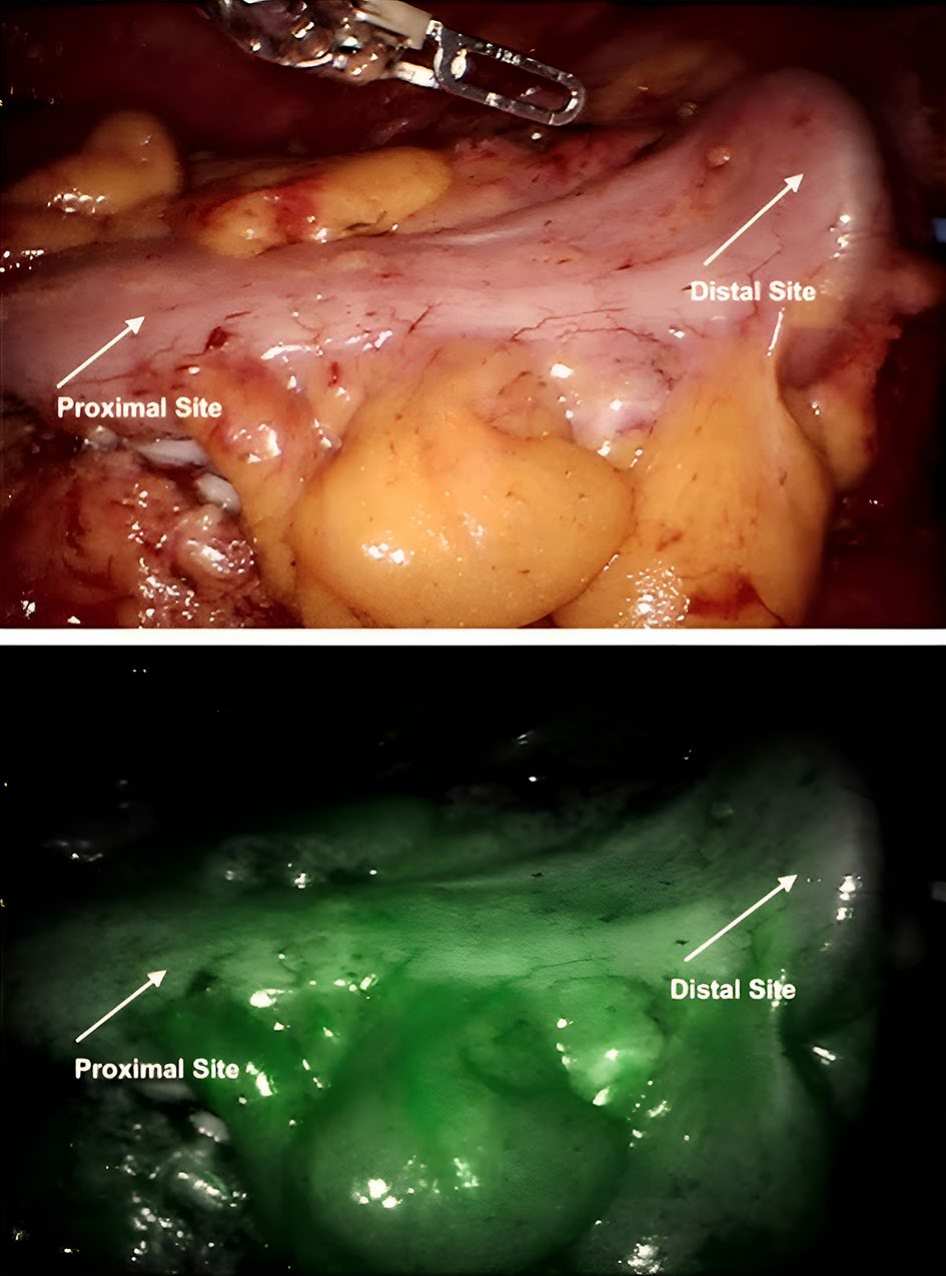
Prepared bowel in white and infrared light + ICG, respectively

After bowel resection and placement of the circular stapler anvil in the oral end, the bowel segment was placed intraabdominally to obtain full visibility of the bowel serosa and a distance to the camera tip of 5 cm (Fig. 1). The perfusion was only assessed at this time before connecting the anvil to the base of the stapler and performing the anastomosis.

To investigate whether dosage impacted our assessment, patients randomly received either 7.5 mg or 15 mg ICG *(VERDYE; Diagnostic Green GmbH, Germany)* as an i.v. bolus. At the same time, the camera was switched to near-infrared light (*Firefly, da Vinci Robotic Assisted Surgical System, Intuitive Inc, CA, USA*.), and a stopwatch was started. With ICG injection as time zero, the fluorescence perfusion in the proximal part of the anastomosis was evaluated by the following parameters: “Time to visibility” (TTV), “time to maximum visibility” (TMV), and “time to wash-out” (TWO) as judged visually by the surgeon and recorded intraoperatively. In addition, the surgeons were asked to decide whether they considered blood supply to be sufficient to perform an anastomosis or whether they would do a re-resection.

The ICG-FI was video recorded, and anonymized videos were exchanged between centers and surgeons for interobserver variation analysis and software analysis. None of the surgeons analyzed videos from their own department.

The videos from the ICG-FI procedures were analysed using a validated pixel analysis software (q-ICG) as previously described [32–36]. This software was developed to quantify the fluorescence using the normalized slope from the fluorescence intensity time curve as an indirect measure of blood flow. This method showed a good correlation to another method of measuring tissue blood flow with the injection of radioactive marked microspheres [32]. The software program was developed on basis of experimental studies on pigs.

**Fig. 2:**
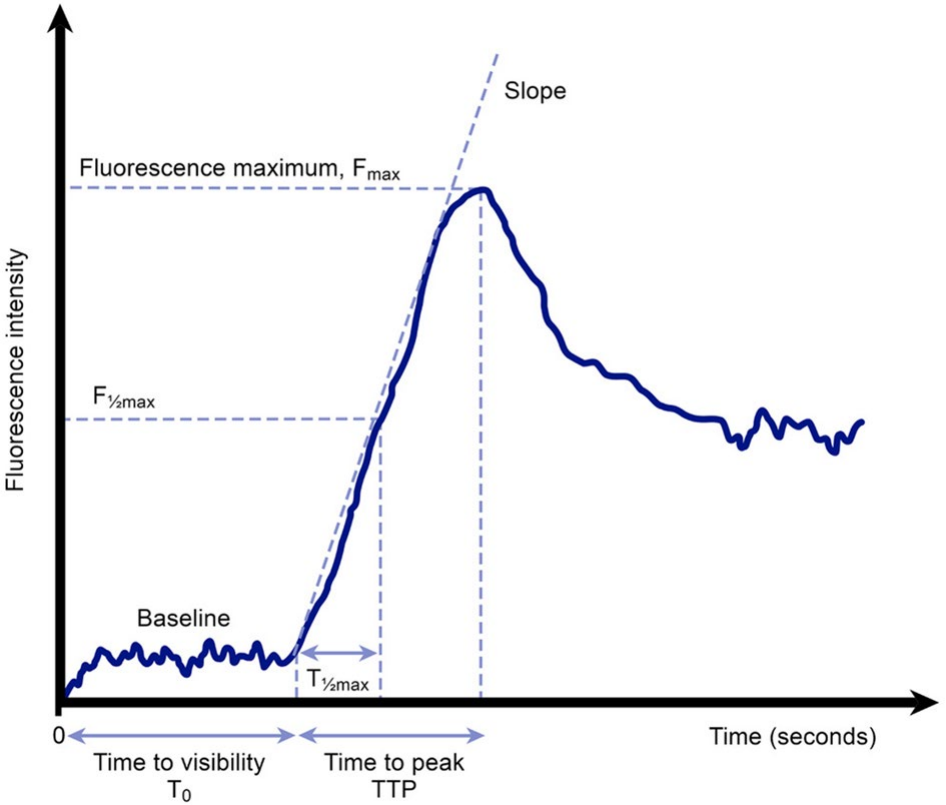
Fluorescence time curve. Normalized Slope is defined as fluorescence increase over time divided by maximum fluorescence minus baseline fluorescence

The normalized slope is defined as fluorescence increase over time divided by maximum fluorescence minus baseline fluorescence (∆Fluorescence intensity/∆time)/(maximum intensity–baseline intensity) (Fig. 2). Regions of interest (ROI) near the anvil (distal) and 5 cm orally (proximal) to the edge of the anvil were chosen (Fig. 1), and pixel analysis was performed in these ROIs.

### Data sources

Clinical baseline characteristics were obtained pre-operatively from the patient record. Intra-operatively the surgeon and nurse recorded the requested data for the ICG-FI in collaboration. Mean arterial blood pressure during the procedure was recorded. Complications within 30 days post-operatively were recorded and graded according to the Clavien-Dindo classification. In addition, any recognized clinical AL, grades B, and C were recorded and defined according to the definition given by the International Study Group of Rectal Cancer [37].

Postoperatively, the videos were, to simulate real-life, presented only once and continuously by peer surgeons.

All data were entered into a RedCap database, provided by OPEN, *Open Patient data Explorative Network.* Data were recorded by single entry.

### Statistics

Bland–Altman (BA) plots were used to evaluate interobserver variation. The two-sample *t*-test for numerical variables and Fisher's exact test for categorical variables were used to compare the background variables between the groups with AL and non-AL. Values for TTV, TMV, and TWO in the two groups were presented as Kaplan–Meier curves and compared by log-rank test. The time differences were evaluated by estimating hazard ratios (HR) comparing the AL group with the non-AL group by Cox regression, both unadjusted as well as adjusted for age, comorbidity, performance status, use of tobacco and alcohol, mean arterial blood pressure, and ICG dosage.

A Wilcoxon rank-sum test was used for the pixel analysis to compare the groups with AL and non-AL and presented by median and interquartile range (IQR).

Statistical calculations were performed using STATA software (version 16; Stata Corp LP, College Station, Texas, USA). P-values less than 0.05 were considered statistically significant.

### Ethics and approvals

The study was approved by the Regional Scientific Ethical Committees of Southern Denmark (Project-ID: S-20160137) and the Danish Data Protection Agency (jr.no. 16/30618).

All patients received written and orally information about the project and written consent was obtained from each patient.

### Results

Fifty-five patients were included, and five patients developed clinical AL (9.1%). We found significantly more patients with heart disease (*p* = 0.018), diabetes (*p* = 0.001), and lymph node metastases (*p* = 0.048) in the AL group. There were no significant differences in other baseline characteristics between the AL and non-AL group (Table 1).

**Table 1:** Baseline characteristics for patients with and without anastomotic leakage (AL)

	No leak = 50	Leak = 5
Male Female	31 (62%) 19 (38%)	5 (100%) 0 (0%)
Age, mean (SD)	63.9 (8.6)	70 (6.4)
BMI, mean (SD)	26.0 (4.0)	26.1 (3.3)
ASA 1 ASA 2	19 (38%) 31 (62%)	0 (0%) 5 (100%)
*Heart disease* (*p* = 0.018): No Yes Unknown	45 (90%) 4 (8%) 1 (2%)	2 (40%) 3 (60%) 0 (0%)
*Diabetes *(*p* = 0.001) No Yes Unknown	48 (96%) 1 (2%) 1 (2%)	2 (40%) 3 (60%) 0 (0%)
cT0 cT1 cT2 cT3 cT4 cTx	1 (2%) 6 (12%) 16 (32%) 19 (38%) 4 (8%) 4 (8%)	0 (0%) 0 (0%) 2 (40%) 2 (40%) 1 (20%) 0 (0%)
*Positive Lymph Nodes* (*p* = 0.048) cN0 cN1 cN2 cNx	21 (42%) 13 (26%) 7 (14%) 9 (18%)	0 (0%) 4 (80%) 1 (20%) 0 (0%)
Operation time, mean (SD), minutes	234.40 (84.81)	284.00 (72.66)
Bleeding ml, mean (SD)	59.91 (62.37)	60 (22.36)
Anastomotic distance from the anal verge, mean, cm (SD)	7.93 (4.75)	5.60 (2.70)
Drain No Yes	6 (70%) 15 (30%)	2 (40%) 3 (60%)
Diverting loop-ileostomy No Yes	30 (60%) 20 (40%)	2 (40%) 3 (60%)
No. of transverse staplings 1 2 3 Unknown	17 (34%) 26 (52%) 4 (8%) 3 (6%)	1 (20%) 3 (60%) 0 (0%) 1 (20%)

*SD* Standard Deviation, *BMI* Body Mass Index, *ASA *American Society of Anesthesiologists, *cT *clinical Tumor stage, *cN *clinical Lymph Node metastasis

### Visual assessment

For the inter-observer investigation, 11 of 55 patients were excluded due to difficulty in video transfer. Due to missing observer data, only 43, 40, and 40 recordings were included in the analysis of TTV, TMV, and TWO, respectively. Missing data occurred both in the intra-operative group and in the observer group. One patient in the observer group developed AL.

**Fig. 3:**
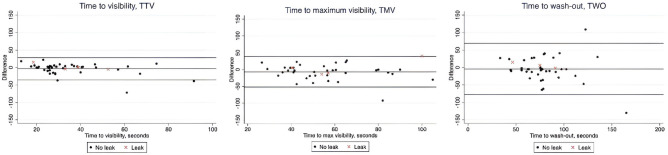
Bland Altman plots illustrating interobserver variation in the leak and no-leak patients assessed intra-operatively by surgeons and postoperatively by peer surgeons (video recordings). The closer dots are to the centerline, the more agreement between the primary surgeon and blinded observer

We found poor agreement for all three parameters, TTV, TMV, and TWO, and the observations by the operating surgeon and the blinded observer differed by up to 70 s. The inter-observer variation was lowest for TTV and highest for TWO (Fig. 3).

In all the operated cases the surgeon considered the blood perfusion to be sufficient to construct an anastomosis based on the ICG-FI, and did not consider a re-resection. Forty-four videos were available for postoperative assessment of whether a re-resection would be recommended, and in seven cases the observer would recommend a re-resection based on the visual assessment of the ICG-FI, but only two of these patients developed an AL. Thus, the postoperative visual assessment of ICG-FI could predict AL came out with a sensitivity of 40% (95% CI: 0.05;0.85) and a specificity of 87% (95% CI 0.73;0.96).

**Fig. 4:**
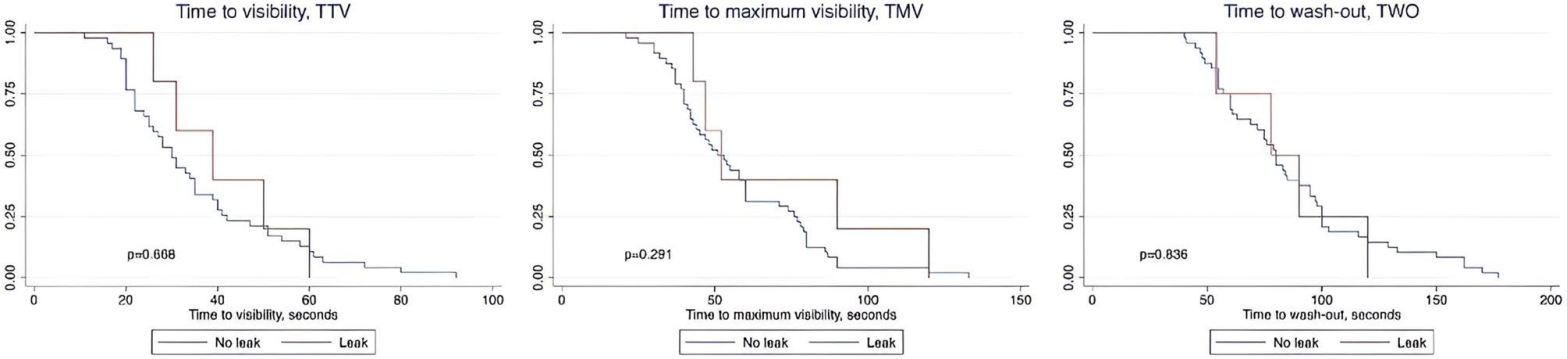
Kaplan–Meier curves illustrating the subjective analysis in the leak and no-leak patients. Number of patients included in the subjective analyses of ICG for TTV, TMV, and TWO was 52, 53, and 52, respectively

The number of patients included in the intraoperative subjective analyses of ICG for TTV, TMV, and TWO was 52, 53, and 52 s, respectively. Comparing time intervals between AL and non-AL we obtained HRs for TTV 0.82 (95% CI: 0.32, 2.08), for TMV 0.62 (95% CI 0.24, 1.59), and 1.11 (95% CI 0.40, 3.11) for TWO. There was no significant difference between the leak and the no-leak group (Fig. 4). Adjusting for confounders the HR for leakage was 1.69 (95% CI 0.57, 5.08), 1.09 (95% CI 0.36, 3.35), and 2.15 (95% CI 0.64, 7.16) for TTV, TMV, and TWO, respectively. Adjusting for the number of cross staples did not change the HRs.

### Quantitative assessment

Quantitative pixel analysis with q-ICG showed a significant difference between the leak and the no-leak group when looking at the results from the ICG-FI 5 cm proximal from the edge of the anvil. The normalized slope was median 0.08 (0.07;0.10) and 0.13 (0.10;0.17), respectively (*p* = 0.04) (Table 2). We included 31 patients without AL and 5 patients with AL in this analysis. Thirteen videos were excluded from this analysis due to poor pixel quality, which compromised the software analysis.

**Table 2:** Quantitative perfusion analyses, normalized slope (∆Fluorescence intensity/∆time)/(maximum intensity – baseline intensity)

	No Leak	Leak n = 5	*p*-value	Dosage 7.5 mg *n* = 20	Dosage 15 mg	*p*-value
Perfusion distally Median (IQR)	*n* = 31 0.13 (0.10;0,15)	0.07 (0.05;0.13)	0.10	0.14 (0.11;0.16)	n = 16 0.11 (0.07;0.14)	0.07
Perfusion proximally Median (IQR)	*n* = 30 0.13 (0.10;0.17)	0.08 (0.07;0.10)	**0.04**	0.13 (0.10;0.16)	*n* = 15 0.12 (0.09;0.16)	0.53

Distally = anastomotic site (anvil), proximally = 5 cm proximal to the anastomotic site; Statistics: Wilcoxon Rank sum test. Bold value is statistically significant (*p* < 0.05)

### ICG dose

There was no difference in TTV, TMV, or TWO when comparing patients receiving low or high doses of ICG (Table 3), regardless of whether the subjective evaluation was intraoperative or based on video recordings. Similarly, we found no difference in the normalized slope/fluorescence intensity when using a low or high ICG dose (Table 2).

**Table 3:** Subjective evaluation by surgeon and observer of ICG-FI in time to visibility (TTV), time to maximum visibility (TMV) and time to wash-out (TWO) from the two different ICG dosages

Dose of ICG	Primary surgeon			Observer		
	7.5 mg	15 mg	*p*-value	7.5 mg	15 mg	*p*-value
TTV	32 (26–38)	33 (26–40)	0.71	29 (23–34)	40 (30–39)	0.08
TMV	51 (42–59)	57 (45–69)	0.44	51 (54–78)	66 (54–78)	0.11
TWO	70 (59–81)	82 (69–95)	0.2	69 (57–82)	85 (72–98)	0.09

Values are mean 95% confidence limits in brackets

### Discussion

In this study, we chose to assess the perfusion after resection and placement of stapler anvil before creating the anastomosis to mimic the clinical situation where the surgeon must decide whether the blood flow is sufficient, or a further resection is necessary before creating the anastomosis. A post-anastomotic analysis may be relevant but meets the problem with the washout period of ICG which may impair interpretation. At least for the non-software-based visual analysis.

In our study we found no difference in the subjective assessment of ICG-FI or qICG using 7,5 mg of ICG or 15 mg of ICG (Table 2). Different dosages have been used varying from a bolus of 6 mg to a bolus of 0.5 mg/kg. No consensus on dosage has been achieved, but titration according to weight might be reasonable.

Most studies exploring fluorescence imaging in gastro-intestinal surgery have been based on the assessment of perfusion on subjective appraisal [30]. A recent meta-analysis [38] reported and found overall frequency of AL on 6.7%, with 4.2% in the ICG group compared to 11.3% in the control group and the difference was highly significant. Due to the heterogeneity of the included studies, they carefully conclude that ICG fluorescence imaging appears to be a promising tool to reduce the rate of AL. In our study we were not able to predict AL from the subjective assessment of ICG-FI.

We found wide inter-observer variation in all parameters chosen for subjective visual evaluation of ICG-FI. None of the patients had a re-resection performed based on the results of ICG-FI, but five of the patients developed AL. In seven patients, the blinded observer would recommend re-resection, but only two of these patients developed AL. Based on this, we found poor sensitivity (40%) of the visual ICG-FI in identifying patients who develop AL, and we suggest that subjective semiquantitative evaluation of ICG-FI should be explored and refined further.

A randomized study [22] including 377 patients, found an overall incidence of AL of 12.7%, with 9.1% in the ICG group and 16.3% in the non-ICG group (*p* = 0.04). There was no difference in AL rate for high anastomoses (9–15 cm from the anal verge), but a significant difference in low anastomoses (14.4% vs 25.7%). This difference consisted of more grade A leakages in the non-ICG group. Perfusion was assessed visually and defined as good if there were uniform fluorescence within 2–3 min.

Another randomized study [23] including 240 patients from three hospitals AL occurred in 7% in total with 5% in the ICG group, and 9% in the non-ICG group. Perfusion was evaluated visually as good, poor, or absent within 1 minute after ICG injection.

In our study, surgeon-observer agreement was better in the early phase of fluorescence, when intensity was highest and became more divergent as time passed (Fig. 2). This emphasizes the importance of fluorescence assessment over a short time-period, for example 1 min.

In the two studies, impaired perfusion in the ICG groups was seen in 19.2% [22] and 11% [23], respectively, leading to further resection. In none of the 55 patients in our study the surgeons considered further resection. The risk of futile resections is an important problem, which hasn't been devoted much attention in any of the previous studies.

Software programs have been developed and validated for the quantification of bowel blood perfusion with ICG-FI [27, 32–36]. By utilizing our quantification software (q-ICG), we found a significant difference between the leak and no-leak patients when measuring 5 cm proximal to the anastomotic site. Due to our small sample size with only five events of anastomotic leakages, this difference should be interpreted with caution and evaluated further in a larger setup.

### Strengths and limitations

The present study has several limitations. The patient cohort was limited to 55 patients, of whom only five had AL.

We performed the ICG-FI after the anvil was placed, which might entail temporary compression on blood vessels at the anastomotic site, with the risk of a false low fluorescence intensity. This compression ceases once the anastomosis is performed, and a second fluorescence evaluation at this time might have given other results.

Conventional ICG-FI as used in our study only reflects the serosa side of the bowel. It has been shown that the mucosa is more susceptible to ischaemia than the serosa [39]. Therefore, it would be interesting to assess the fluorescent intensity from the mucosal side with trans-anal ICG-FI [40].

The strength of our study was the systematic approach towards evaluating the ICG-FI and its comparison with computer-based pixel analysis. We did not find any relationship between the results and the infusion of 7,5 mg or 15 mg.

All the qICG values reported were from a postoperative examination of video recordings of the ICG angiography during the operation. We did not have real-time pixel analysis software available at the time of the study. Since then, on table qICG pixel analysis is now feasible on a touch screen tablet, where the surgeon can define areas of interest intraoperatively, for example, anastomotic site [41]. In the future it will be essential to establish quantitative fluorescence cut-off values, where AL due to poor perfusion might be prevented by re-resection [42].
